# Preventive effect of *Lactobacillus fermentum* Lee on activated carbon-induced constipation in mice

**DOI:** 10.3892/etm.2014.2064

**Published:** 2014-11-11

**Authors:** YU QIAN, HUAYI SUO, MUYING DU, XIN ZHAO, JIAN LI, GUI-JIE LI, JIA-LI SONG, ZHENHU LIU

**Affiliations:** 1 Department of Biological and Chemical Engineering, Chongqing University of Education, Chongqing 400067, P.R. China; 2 College of Food Science, Southwest University, Chongqing 400715, P.R. China; 3 Institute of Qinghai-Tibetan Plateau, Southwest University for Nationalities, Chengdu, Sichuan 610041, P.R. China; 4 Department of Food Science and Nutrition, Pusan National University, Busan 609-735, Republic of Korea; 5 Xinjiang Academy of Agricultural Sciences, Urumqi, Xinjiang 830091, P.R. China

**Keywords:** *Lactobacillus fermentum* Lee, activated carbon, constipation, bisacodyl, gastrointestinal transit

## Abstract

The aim of this study was to investigate the effects of *Lactobacillus fermentum* Lee (LF-Lee) on activated carbon-induced constipation in ICR mice. ICR mice were orally administered lactic acid bacteria for nine days. Body weight, dietary and water intake, defecation status, gastrointestinal (GI) transit and defecation time, as well as levels of motilin (MTL), gastrin (Gas), endothelin (ET), somatostatin (SS), acetylcholinesterase (AChE), substance P (SP) and vasoactive intestinal peptide (VIP) in serum were measured to evaluate the preventive effects of LF-Lee on constipation. Bisacodyl, a laxative drug, was administered as a positive control. The time taken until the first defecation of a black stool for normal, control, bisacodyl- (100 mg/kg, oral administration), *Lactobacillus bulgaricus* (LB)-, LF-Lee low dose (L)- and LF-Lee high dose (H)-treated mice was 90, 218, 117, 180, 161 and 151 min, respectively. Following the consumption of LB, LF-Lee (L) or LF-Lee (H), or the oral administration of bisacodyl, the GI transit was reduced to 55.2, 65.8, 73.1 and 94.6%, respectively, of the transit in normal mice. The serum levels of MTL, Gas, ET, AChE, SP and VIP were significantly increased and those of SS were reduced in the mice treated with LF-Lee compared with those in the untreated control mice (P<0.05). These results demonstrate that lactic acid bacteria have preventive effects on constipation in mice and that LF-Lee has superior functional activity.

## Introduction

Yak yogurt is a traditional dairy product from the Qinghai-Tibet Plateau. Rich in nutrients, it aids digestion, stimulates appetite and also has antibacterial, pore constringing, sedation and hypnosis effects. In addition, it can also be beneficial in patients with chronic mild diarrhea (1). Furthermore, it has also been found to reduce levels of cholesterol, prevent arteriosclerosis and cancer, and avoid premature aging (2). The authors of the present study have separated microorganisms from yak yoghurt prepared by Tibetan inhabitants and identified lactic acid bacteria, which have been named *Lactobacillus fermentum* Lee (LF-Lee).

Constipation is a common clinical condition that involves a reduction in the frequency of defecation, the quantity of feces, dry stools and defecation difficulties. When activated carbon is administered to mice, constipation occurs due to a reduction in the quantity of gastrointestinal (GI) fluid and weakening of GI peristalsis as the activated carbon is absorbed on the surface of the GI mucosa, which reduces GI functioning. Therefore, numerous studies have carried out experiments on animal models with activated carbon-induced constipation (3). Previous studies have used the activated carbon-induced constipation model to demonstrate the effectiveness of drugs for constipation treatment (4,5). In addition, one study revealed that a megadose of activated carbon resulted in digestive tract obstruction (6).

In the present study, the functional effects of LF-Lee in the alimentary tract were examined using an activated carbon-induced mouse model of constipation. The GI transit, time taken until the first defecation of a black stool, and serum assays of levels of motilin (MTL), gastrin (Gas), endothelin (ET), somatostatin (SS), acetylcholinesterase (AChE), substance P (SP) and vasoactive intestinal peptide (VIP) were examined. Bisacodyl was used as a positive control. Bisacodyl is a drug routinely used in the treatment of acute and chronic constipation. It works by stimulating the rectal nerve endings to promote bowel motility. The laxity helps to treat constipation (7–9).

With LF-Lee as the subject, the present study took *Lactobacillus bulgaricus* (LB), which is used in yoghurt production, as a comparative strain. By comparing the bile tolerance and hydrophobic properties of LF-Lee and LB, the ability of LF-Lee to pass through the stomach and guts and adhere to the small intestine was studied. This allowed further study of its curative effect to constipation, thus laying a scientific foundation for the development of lactic acid bacteria.

## Materials and methods

### Microorganism strains

LF-Lee was separated and identified from yak yoghurt produced in the Hongyuan Grassland (Ngawa Prefecture, China) and deposited at the China Center for Type Culture Collection (CCTCC, Wuhan, China), bearing CCTCC Accession Number M2013512. LB was purchased from the Institute of Microbiology of the Chinese Academy of Sciences (Beijing, China).

### Animals

Seven-week-old female ICR mice (n=120) were purchased from the Experimental Animal Center of Chongqing Medical University (Chongqing, China). The mice were maintained in a temperature- and humidity-controlled (temperature 25±2°C, relative humidity 50±5%) facility with a 12-h light/dark cycle and free access to a standard rat chow diet and water. The present study was approved by the Ethics Committee of ChongQing Medical University.

### Endurance capacity of lactic acid bacteria to artificial gastric juice of pH 3.0

Artificial gastric juice was prepared using 0.2% NaCl and 0.35% pepsin, adjusted to pH 3.0 and then vacuum-filtered to remove bacteria on a clean bench. A total of 5 ml reactivated bacterial culture was centrifuged at 1,006 × g for 10 min. The bacterial pellet was collected and re-suspended in 5 ml sterile saline, following which 1 ml suspension was mixed with 9 ml artificial gastric juice and incubated in a thermostatic oscillator at 37°C and 300 rpm. Subsequently, 200 μl sample was pipetted at 0 and 3 h, poured onto a plate with de Man, Rogosa and Sharpe (MRS) agar and then incubated at 37°C for 48 h. The number of colony-forming units (CFUs) was counted and the survival rate was determined, as previously described (10).

### Determination of the bacterial tolerance to bile salt (ox gall) of different concentrations

A total of 100 μl reactivated bacterial culture was inoculated at 2% (v/v) into MRS-thio (MRS + 0.2% sodium thioglycolate) broth which contained 0.0 (control), 0.3, 0.5 and l.0% ox gall (Shanghai Ekear Bio&Tech Co., Ltd., Shanghai, China), respectively. Following incubation at 37°C for 24 h, the optical density (OD) value of each culture was measured and the tolerance of the bacterial strain to ox gall was determined by comparing the OD of the ox gall tube with that of the control tube (10).

### Determination of the hydrophobic properties of lactic acid bacteria

Reactivated bacterial culture (5 ml) was centrifuged at 1,370 × g for 10 min. The bacterial pellet was collected, resuspended in 5 ml phosphate-buffered saline (PBS; 50 mM; pH 6.5) and centrifuged at 1,370 × g for 10 min; the process was then repeated. Using PBS as a blank for absorption, the final bacterial suspension was adjusted using PBS to produce a 1.00 absorbance (A_0_) at 560 nm. A total of 4 ml of the adjusted bacteria suspension was added to 0.8 ml dimethylbenzene, vibrated for 30 sec and allowed to settle into layers. The aqueous layer was measured for absorbance (A) at 560 nm (blank: PBS) and the results were recorded (11).

### Induction of constipation in mice

To investigate the preventive effects of LF-Lee against activated carbon-induced constipation, the animals were divided into six groups with 20 mice in each group. The experimental design was as follows: the normal and control groups received a normal diet for nine days; the LF-Lee high dose (H), LF-Lee low dose (L) and LB groups were orally administered a 2-ml dose of concentration 1×10^9^, 1×10^8^ and 1×10^9^ CFU/ml, respectively; the drug cure group mice were treated with a 100 mg/kg dose of bisacodyl dissolved in water for nine days. The control and treatment groups received an oral administration of activated carbon (0.2 ml 10% activated carbon, w/w; activated carbon dissolved in 10% gum arabic) at 18:00 from the sixth to the ninth day to induce constipation (12). The body weight, dietary and water intake, and stool weight and moisture were measured at 09:00 every day.

### Measurement of the defecation status of the mice

This measurement was performed to determine whether the prokinetic actions of the lactic acid bacteria were capable of propagating a prokinetic signal along the entire length of the GI tract. The excreted fecal pellets of individual mice were collected daily at 09:00 for the duration of the experiment. The total number, weight and water content of the pellets were determined. The water content was calculated as the difference between the wet and dry weight of the pellet. After 16 h, the mice in the control and treatment groups received 10% activated carbon and the normal group was administered 10% gum arabic by intragastric gavage. The animals were individually placed into small transparent cages and allowed unrestricted access to food and tap water. The length of time from carbon meal administration to the appearance of darkened feces was recorded. Feces were collected, counted, evaluated for water content and weighed following the intragastric gavage administration.

### GI transit and defecation time

Mice were fasted for 16 h from the ninth day at 18:00; however, they were not deprived of water. After 16 h, the mice in the control and treatment groups received an oral administration of 10% activated carbon while the mice in the normal group received an oral administration of 10% gum arabic. After 30 min, the mice were sacrificed by cervical dislocation under anesthesia with diethyl ether. A total of 10 mice in each group were dissected and the small intestine, from the pylorus to the cecum, was carefully removed. The GI transit of each mouse was calculated as the percentage of the distance traveled by the activated carbon meal relative to the total length of the small intestine. The following equation was used to calculate GI transit: GI transit (%) = distance traveled by the activated carbon/total length of the small intestine × 100. The remaining 10 mice from each group were used to measure the time taken to the first defecation of a black stool following oral administration of 10% activated carbon.

### Levels of MTL, Gas, ET, SS, AChE, SP and VIP in the serum

The levels of MTL, Gas, ET, SS, AChE, SP and VIP in the serum were determined using radioimmunoassay kits (Beijing Puer Weiye Biotechnology Co., Ltd., Beijing, China). The serum was collected from the heart following surgery.

### Statistical analysis

Data are presented as mean ± standard deviation. Differences between the mean values for individual groups were assessed using a one-way analysis of variance (ANOVA) with Duncan’s multiple range test. P<0.05 was considered to indicate a statistically significant difference. SAS 9.1 (SAS Institute Inc., Cary, NC, USA) was used to carry out the statistical analyses.

## Results

### Biological barrier resistance and hydrophobic properties of lactic acid bacteria

The GI survival abilities of the lactic acid bacteria were evaluated using synthetic gastric juice, bile salt and hydrophobic property tests. LF-Lee revealed higher GI survival abilities than LB (Table I). In particular, in the different concentrations of bile salt, the growth of LF-Lee was 10–12-fold higher than that of LB.

### Body weight during the experiment

**Figure 1:**
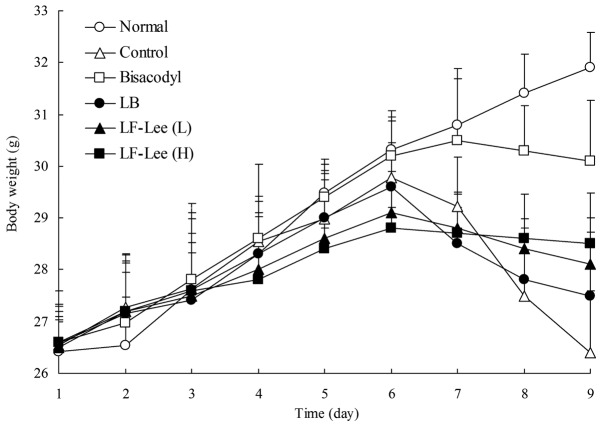
Body weights of mice during the experiment (n=10 per group). The dose of bisacodyl was 100 mg/kg body weight (bw). LB, *Lactobacillus bulgaricus* [1.0×10^9^ colony-forming unit (CFU)/kg bw]; LF-Lee (L), *Lactobacillus fermentum* Lee low dose (0.5×10^9^ CFU/kg bw); LF-Lee (H), *Lactobacillus fermentum* Lee high dose (1.0×10^9^ CFU/kg bw).

Body weight is an important marker of constipation in mice; the body weights of mice with activated carbon-induced constipation are lower compared with those of normal mice (12). Bisacodyl is an effective medicine for constipation treatment and it was used as a positive control in the present study. The normal mice had a normal diet and their body weight increased during the experiment. The body weight of the control mice with activated carbon-induced constipation was significantly decreased after six days. As shown in Fig. 1, following the initiation of activated carbon-induced constipation, the body weights of the mice in the LF-Lee and LB groups were significantly lower compared with those in the normal and bisacodyl-treated groups; however, they were higher than in the activated carbon-induced constipation control mice. LF-Lee treatment of the mice was able to alleviate the weight loss more effectively than LB treatment.

### Effect of lactic acid bacteria on diet and water uptake

The dietary intake of the mice in each group remained stable from the first to the sixth day of the experiment. From the sixth day following the induction of constipation, the dietary intake of the control and treatment group mice decreased significantly; in particular, the intake of the control group decreased the most. However, the dietary intake of the LF-Lee groups remained higher than that of the LB and control groups, and approached that of the bisacodyl-treated group (Table II). The pattern of the uptake of drinking water by the mice in each group (Table III) was similar to that of the dietary intake. Thus, in the present study, LF-Lee was able to reduce dietary intake loss and thereby relieve anorexia following the induction of constipation.

### Effect of lactic acid bacteria on the defecation status of mice

Constipation refers to bowel movements that are infrequent or hard to pass. Thus, pain in the abdomen and bloating are characteristics of constipation. There are a number of causes of constipation, including medication, poor bowel habits, a diet low in fiber, abuse of laxatives, hormonal disorders and disease that may be primarily of other parts of the body but that also affects the colon. Therefore, defecation is the most important criterion of constipation. The current study divided defecation status into three parts: i) defecation weight (g) which was observed as a main point to estimate the constipation situation; if the defecation weight was heavy, the mice had positive defecation qualities; ii) particle counts of defecation (pieces), where a greater number of pieces defecated implied that mice had good GI movement; iii) water content of defecation, where a higher water content indicated improved stool qualities. From the first to the sixth day, the defecation weight (g), particle counts of defecation and water content of defecation (%) in each group were not significantly different. However, the defecation weight and particle counts of defecation in the bisacodyl-treated group were slightly higher compared with those in the other groups (Table IV). From the seventh to the ninth day, the defecation weight, particle counts of defecation and water content of defecation decreased to 0.37 g, 19 pieces and 16%, respectively, in the control group. Defecation weights and particle counts of defecation decreased to 0.74 g/38 pieces, 0.40 g/21 pieces, 0.55 g/26 pieces and 0.61 g/30 pieces, and water content of defecation to 40, 23, 27 and 34% in the bisacodyl, LB, LF-Lee (L) and LF-Lee (H) groups, respectively. These results demonstrated that the LF-Lee groups had an improved defecation status compared with the control group following induced constipation and, thus, that LF-Lee may be used as a potential treatment for constipation.

### Time taken to the first defecation of a black stool

**Figure 2:**
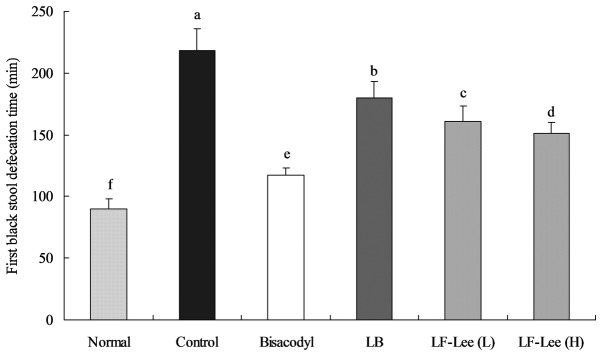
First black stool defecation time of mice in the various groups on the final day of the experiment, following the induction of constipation by activated carbon (n=10 per group). The dose of bisacodyl was 100 mg/kg body weight (bw). LB, *Lactobacillus bulgaricus* [1.0×10^9^ colony-forming unit (CFU)/kg bw]; LF-Lee (L), *Lactobacillus fermentum* Lee low dose (0.5×10^9^ CFU/kg bw); LF-Lee (H), *Lactobacillus fermentum* Lee high dose (1.0×10^9^ CFU/kg bw); ^a–f^Mean values with different letters over the bars are significantly different (P<0.05) according to Duncan’s multiple range test.

The time taken to the first defecation of a black stool in each group of mice following the administration of activated carbon, is shown in Fig. 2. The defecation time was shortest (90±8 min) in the normal group and longest (218±18 min) in the control group. The defecation time in the bisacodyl-treated group (117±6 min) was higher compared with that in the normal group. The time taken to the first defecation of a black stool in the LB, LF-Lee (L) and LF-Lee (H) groups was 180±13, 161±12 and 151±9 min, respectively. According to the results of the defecation times, LF-Lee revealed a stronger effect than LB as an inhibitor of constipation.

### GI transit

**Figure 3:**
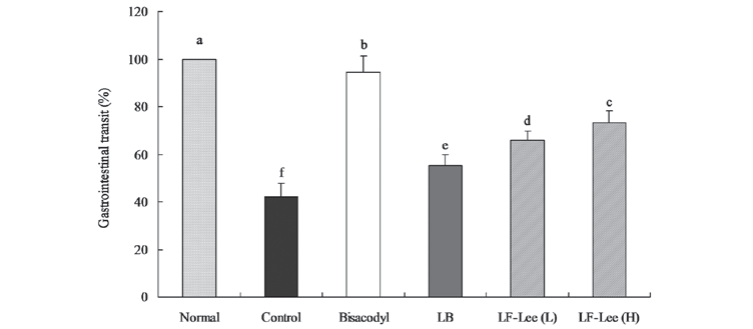
Effect of various treatments on the gastrointestinal (GI) transit in the mouse model of activated carbon-induced constipation (n=10 per group). The dose of bisacodyl was 100 mg/kg body weight (bw). LB, *Lactobacillus bulgaricus* [1.0×10^9^ colony-forming unit (CFU)/kg bw]; LF-Lee (L), *Lactobacillus fermentum* Lee low dose (0.5×10^9^ CFU/kg bw); LF-Lee (H), *Lactobacillus fermentum* Lee high dose (1.0×10^9^ CFU/kg bw); ^a–f^Mean values with different letters over the bars are significantly different (P<0.05) according to Duncan’s multiple range test.

The constipation-inhibiting effects of the treatments were determined by GI transit in mice following the administration of activated carbon (0.2 ml/mouse, 10% activated carbon). In the bisacodyl-treated group, the mean GI transit was 94.6±6.7%, which was higher than that of the control group (42.1±5.6%; Fig. 3). The GI transits of the LB, LF-Lee (L) and LF-Lee (H) groups were 55.2±4.6, 65.8±4.1 and 73.1±5.1%, respectively. Thus, LF-Lee increased the GI transit compared with that of the control group and thereby reduced constipation and had an increased functional effect on GI transit.

### Levels of MTL, Gas, ET, SS, AChE, SP and VIP in the serum

Normal mice demonstrated the highest levels of MTL, Gas, ET, AChE, SP and VIP among the groups; these levels were significantly reduced in the control mice (P<0.05; Table V). The levels of MTL, Gas, ET, AChE, SP and VIP in the bisacodyl-treated mice were similar to those of the normal mice. These levels in the lactic acid bacteria-treated mice approached those in the normal mice but were higher than those of the control mice. The LF-Lee-treated mice demonstrated higher levels of MTL, Gas, ET, AChE, SP and VIP compared with the LB-treated mice. Furthermore, the high dose LF-Lee-treated mice demonstrated similar levels of these substances to the normal and bisacodyl-treated mice. The levels of SS in mice showed the opposite tendency.

## Discussion

Patients with constipation frequently present with anorexia (13). Therefore, dietary and water intake can be taken as standards to measure the severity of the constipation. Furthermore, patients with constipation also present with symptoms such as the weakening of intestinal peristalsis and difficulty in defecation (14,15). When food is digested, the indigested content forms a stool. However, as intestinal peristalsis slows down, the rate of the digestive movement of the stool becomes slow. Once the movement of the stool slows down, more water is absorbed from the stool. The stool subsequently hardens and dries (16). Therefore defecation time, stool quantity and water content in the stool may all be used as standards to measure constipation.

Probiotics, including lactic acid bacteria, are usually present in food or are taken as oral supplements. They survive the strong acidic conditions in the stomach and upper intestinal tract and upon reaching their final destination (usually the large intestine), colonize and give play to their physiological efficacy (17). Therefore, in order to study whether probiotics are able to pass the through stomach and intestine to colonize the intestinal tract, external experiments to model the conditions in the intestinal tract were conducted. In these, the growth of lactic acid bacteria was analyzed in order to measure the endurance of the bacteria during a screening experiment. The potential probiotic function of bacteria may be identified by measuring their acid resistance, bile tolerance and hydrophobic properties (10). In the present study, LF-Lee revealed improved qualities of acid resistance, bile tolerance and hydrophobic properties compared with the common lactic acid bacteria, LB. These qualities indicate that the functional effects of LF-Lee may be suitable for humans.

The surviving bacteria passing through the stomach may contact bile acids, such as cholate, in the small intestine. The cholate tolerance of lactic acid bacteria is taken as a standard when identifying potential probiotics (18). In addition to being able to withstand cholate in the small intestine, lactic acid bacteria should be able to adhere to the mucous membrane of the small intestine. Therefore, the hydrophobic properties of lactic acid bacteria are taken as another standard (19). In experiments designed to imitate the environment in the stomach and guts, the tolerance of probiotics to gastric juice and cholate were investigated. The present study revealed that LF-Lee has a stronger tolerance to gastric juice and cholate than the common lactic acid bacteria LB. Thus, LF-Lee is likely to have a higher survival rate upon entering the stomach and guts, with the majority of the bacteria reaching the small intestine. Furthermore, the experiment testing the hydrophobic properties of the bacteria in the current study indicate that LF-Lee is likely to adhere more strongly than LB to the walls of the small intestine and, thus, that a greater number of LF-Lee bacteria should be able to take effect in the small intestine.

During constipation, excrement remains in the small intestine for an abnormally long period of time. Harmful bacteria may consume this excrement and proliferate, thus threatening the health of the intestines and compounding the constipation. Other organs may suffer from damage if the intestines absorb the harmful substance produced by these bacteria (20). Following constipation, the small intestine is alkaline and lactic acid bacteria produce large amounts of acid to adjust the pH value, thus making the environment in the intestines disadvantageous to the growth of harmful bacteria (21). Lactic acid bacteria may also promote GI tract movement and produce active material beneficial to the intestine, thereby preventing further constipation.

Compared with patients with constipation, MTL, Gas, ET, AChE, SP and VIP levels in the serum of healthy individuals are higher whilst the levels of SS are lower (22–24). MTL is a type of GI hormone. It is able to promote intestinal peristalsis and contraction of the intestinal smooth muscle, thus hastening the passing of intestinal contents through the intestinal tract. Gas is a GI hormone that is able to promote GI movement by stimulating GI secretion and stomach contraction. Furthermore, it can also promote contraction of the pyloric sphincter (22). ET is an important element that adjusts cardiovascular functions. Maintaining levels of ET not only prevents acute cardiovascular and cerebrovascular diseases induced by constipation but may also help patients with cardiovascular and cerebrovascular diseases avoid aggravation as a result of constipation (28). However, SS can stimulate the secretion of gastric acid and inhibit the release of pepsin and gastrin, thus aggravating constipation. The release of AChE may help promote the growth of nervous tissue, avoid enteric neuromuscular disease, which is a type of colonic pseudoobstruction, and prevent the development of constipation (28). SP is a type of excitatory neuropeptide that is able to stimulate bowel motility; reductions in the levels of SP are associated with rectal sensory dysfunction. Furthermore, rectal sensory dysfunction increases rectal compliance leading to constipation (29). VIP is a type of non-cholinergic inhibitory neuropeptide. It can influence bowel motility, as it is able to cause descending relaxation during intestinal peristaltic reflex. If the level of VIP is not sufficiently high, the level of normal enteric nerves is affected, leading to constipation (30).

The aim of the current study was to investigate whether the lactic acid bacteria LF-Lee had a preventive effect against activated carbon-induced constipation in mice. LF-Lee exhibited better qualities of acid resistance, bile tolerance and hydrophobic properties than LB. In the mice treated with LF-Lee, the results demonstrated that the time to the first black stool defecation was only marginally longer than that in mice treated with bisacodyl. The GI transit was longer than that in the control mice and was similar to that in the bisacodyl group. The levels MTL, Gas, ET, AChE, SP and VIP in the LF-Lee-treated mice were higher compared with those in the control and common LB-treated mice and the levels of SS demonstrated the opposite tendency. These results suggest that LF-Lee has a significant preventive effect on activated carbon-induced constipation in mice.

## Figures and Tables

**Table I:** Resistance to biological barriers and the level of hydrophobicity of *Lactobacillus fermentum* Lee (LF-Lee).

Strain	Survival in artificial gastric juice of pH 3.0 (%)	Hydrophobic property (%)	Growth in bile salt (%)		

			0.3%	0.5%	1.0%
LF-Lee	87.99±5.21	68.20±3.72	25.31±2.03	20.17±1.89	15.22±1.14
LB	27.81±3.41	25.56±2.71	2.61±0.34	1.57±0.37	1.31±0.22

Values presented are the mean ± standard deviation. LB, *Lactobacillus bulgaricus*.

**Table II:** Food intake (g) by the various groups of mice during the experiment.

					LF-Lee (x10^9^ CFU/kg bw)	
					
Treatment	Normal	Control	Bisacodyl	LB	0.5	1.0
Day 1	2.69±0.12	2.66±0.17	2.72±0.14	2.74±0.12	2.68±0.20	2.72±0.12
Day 2	2.73±0.13	2.71±0.21	2.77±0.12	2.75±0.16	2.70±0.18	2.77±0.22
Day 3	2.90±0.11	2.84±0.20	2.86±0.20	2.79±0.14	2.82±0.13	2.90±0.15
Day 4	3.04±0.15	3.01±0.16	3.02±0.15	3.00±0.12	3.01±0.21	3.15±0.10
Day 5	3.08±0.18	3.06±0.15	3.07±0.10	3.12±0.11	3.13±0.10	3.17±0.12
Day 6	3.10±0.16	3.09±0.14	3.12±0.11	3.15±0.16	3.15±0.10	3.18±0.18
Day 7	3.14±0.10	2.60±0.22	2.88±0.22	2.63±0.12	2.70±0.15	2.77±0.12
Day 8	3.15±0.13	2.19±0.15	2.78±0.17	2.40±0.15	2.53±0.11	2.62±0.15
Day 9	3.22±0.13	2.02±0.09	2.70±0.14	2.22±0.13	2.37±0.12	2.42±0.11

Values presented are the mean ± standard deviation (n=10 per group). LB, *Lactobacillus bulgaricus*; LF-Lee, *Lactobacillus fermentum* Lee; CFU, colony-forming unit; bw, body weight. The dose of bisacodyl was 100 mg/kg bw and the dose of LB was 1.0×10^9^ CFU/kg bw.

**Table III:** Liquid uptake (ml) by the various groups of mice during the experiment.

					LF-Lee (x10^9^ CFU/kg bw)	
					
Treatment	Normal	Control	Bisacodyl	LB	0.5	1.0
Day 1	6.24±0.20	6.25±0.20	6.24±0.20	6.24±0.12	6.25±0.20	6.25±0.10
Day 2	6.26±0.21	6.28±0.15	6.30±0.20	6.22±0.16	6.25±0.18	6.27±0.17
Day 3	6.27±0.12	6.24±0.16	6.30±0.16	6.26±0.12	6.27±0.15	6.29±0.18
Day 4	6.25±.020	6.25±0.20	6.29±0.15	6.28±0.16	6.30±0.11	6.28±0.13
Day 5	6.32±0.20	6.31±0.15	6.32±0.20	6.30±0.13	6.30±0.22	6.30±0.12
Day 6	6.34±0.20	6.28±0.21	6.34±0.18	6.32±0.10	6.32±0.17	6.31±0.16
Day 7	6.37±0.21	6.14±0.17	6.28±0.15	6.17±0.13	6.22±0.15	6.27±0.15
Day 8	6.38±0.18	5.75±0.18	6.25±0.15	5.93±0.14	6.02±0.15	6.10±0.10
Day 9	6.40±0.22	5.63±0.20	6.21±0.17	5.75±0.12	5.93±0.15	6.04±0.11

Values presented are the mean ± standard deviation (n=10 per group). LB, *Lactobacillus bulgaricus*; LF-Lee, *Lactobacillus fermentum* Lee; CFU, colony-forming unit; bw, body weight. The dose of bisacodyl was 100 mg/kg bw and the dose of LB was 1.0×10^9^ CFU/kg bw.

**Table IV:** Defecation status of the various groups of mice during the experiment.

					LF-Lee (x10^9^ CFU/kg bw)	
					
Treatment	Normal	Control	Bisacodyl	LB	0.5	1.0
Days 1–6^a^						
Defecation weight (g)	0.90±0.09	0.94±0.11	1.13±0.07	0.91±0.05	0.91±0.05	0.89±0.06
Defecation particle counts (n)	35±4	36±7	49±6	36±2	35±5	36±4
Water content of defecation (%)	47±4	47±5	55±5	49±4	46±5	48±4
Days 7–9^b^						
Defecation weight (g)	0.91±0.05	0.37±0.06	0.74±0.15	0.40±0.05	0.55±0.05	0.61±0.05
Defecation particle counts (n)	36±3	19±6	38±5	21±3	26±3	30±5
Water content of defecation (%)	46±5	16±3	40±3	23±2	27±4	34±6

a Treatment alone was administered;

b treatment and activated carbon were administered. Values presented are the mean ± standard deviation (n=10 per group).

LB, *Lactobacillus bulgaricus*; LF-Lee, *Lactobacillus fermentum* Lee; CFU, colony-forming unit; bw, body weight. The dose of bisacodyl was 100 mg/kg bw and the dose of LB was 1.0×10^9^ CFU/kg bw.

**Table V:** Effect of various treatments on serum levels in the mouse model of activated carbon-induced constipation (pg/ml).

					LF-Lee (x10^9^ CFU/kg bw)	
					
Analyte	Normal	Control	Bisacodyl	LB	0.5	1.0
MTL	173.2±12.6^a^	101.3±9.7^f^	155.4±8.7^b^	119.7±.7.7^e^	134.2±7.6^d^	143.7±8.1^c^
Gas	80.2±3.2^a^	44.3±2.7^f^	73.6±2.6^b^	50.2±2.1^e^	59.2±2.2^d^	67.2±2.8^c^
ET	13.9±0.4^a^	7.1±0.3^f^	12.0±0.3^b^	8.4±0.3^e^	9.0±0.3^d^	10.0±0.3^c^
SS	33.2±.1.9^f^	61.8±1.6^a^	40.3±2.0^e^	56.2±1.9^b^	50.0±0.9^c^	45.2±0.7^d^
AChE	31.1±1.2^a^	12.7±0.9^f^	27.8±0.9^b^	15.9±0.8^e^	20.3±1.1^d^	23.6±0.7^c^
SP	63.2±2.8^a^	37.2±1.9^f^	55.3±1.7^b^	41.3±0.5^e^	45.7±0.6^d^	50.3±0.8^c^
VIP	52.3±1.9^a^	30.6±1.0^f^	47.1±1.1^b^	33.6±0.9^e^	38.8±1.0^d^	42.7±0.6^c^

Values presented are the mean ± standard deviation (n=10 per group). LB, *Lactobacillus bulgaricus*; LF-Lee, *Lactobacillus fermentum* Lee; CFU, colony-forming unit; bw, body weight; MTL, motilin; Gas, gastrin; ET, endothelin; SS, somatostatin; AChE, acetylcholinesterase; SP, substance P; VIP, vasoactive intestinal peptide. The dose of bisacodyl was 100 mg/kg bw and the dose of LB was 1.0×10^9^ CFU/kg bw.

a–f Mean values with different letters in the same column are significantly different (P<0.05) according to Duncan’s multiple range test.
